# lncRNA WT1-AS attenuates hypoxia/ischemia-induced neuronal injury during cerebral ischemic stroke via miR-186-5p/XIAP axis

**DOI:** 10.1515/med-2022-0528

**Published:** 2022-07-25

**Authors:** Jianquan You, Fei Qian, Yu Huang, Yingxuan Guo, Yaqian Lv, Yuqi Yang, Xiupan Lu, Ting Guo, Jun Wang, Bin Gu

**Affiliations:** Emergency Department, Taizhou People’s Hospital, Taizhou Pharmaceutical High-Tech Zone, Taizhou 225300, Jiangsu Province, China; Emergency Department, Taizhou People’s Hospital, No. 366 Taihu Road, Taizhou Pharmaceutical High-Tech Zone, Taizhou 225300, Jiangsu Province, China

**Keywords:** wilms tumor 1 antisense RNA, cerebral ischemic stroke, microRNA-186-5p, X-linked inhibitor of apoptosis, oxygen glucose deprivation

## Abstract

This study aimed to investigate the role and mechanism of long non-coding RNA (lncRNA) WT1 antisense RNA (WT1-AS) in cerebral ischemic stroke. The Starbase database and dual-luciferase reporter gene assay were used to analyze the interaction between lncRNA WT1 antisense RNA (lncRNA WT1-AS) and microRNA-186-5p (miR-186-5p). Reverse transcription-quantitative PCR analysis was performed to determine lncRNA WT1-AS and miR-186-5p levels. An oxygen glucose deprivation (OGD)-induced SH-SY5Y cell injury model was established. Cell viability and apoptosis were determined using 3-(4,5-dimethylthiazol-2-yl)-2,5-diphenyl tetrazolium bromide and flow cytometric assays, respectively. Caspase 3 activity was evaluated using a caspase 3 activity detection kit. The results showed that miR-186-5p is a direct target of the lncRNA WT1-AS. In addition, lncRNA WT1-AS levels were downregulated and miR-186-5p levels were upregulated in the blood samples of patients with ischemic stroke and OGD-induced SH-SY5Y cells. WT1-AS overexpression promoted OGD-induced cell viability and reduced the cell apoptosis and caspase 3 activity. However, these effects were reversed by miR-186-5p overexpression. Furthermore, the results demonstrated that the X-linked inhibitor of apoptosis (XIAP) was directly targeted by miR-186-5p. Similarly, transfection with the miR-186-5p inhibitor reduced OGD-induced neuronal damage by upregulating XIAP expression. In conclusion, lncRNA WT1-AS attenuates hypoxia/ischemia-induced neuronal injury in cerebral ischemic stroke through the miR-186-5p/XIAP axis.

## 1 Introduction

Stroke is the leading cause of cerebral dysfunction and mortality worldwide [1]. Approximately 795,000 new cases of stroke are recorded in the United States annually, while more than 140,000 individuals die from stroke [2]. Eighty percent of stroke cases are attributed to ischemic stroke [3]. The main features of ischemic stroke include interruption of blood flow and lack of oxygen and glucose supply in the brain cells, eventually leading to impaired neuronal cell function. Current treatment approaches for stroke mainly focus on stimulating nerve replacement and timely resolving the infarction to limit continuous hypoxic stress-mediated damage, which eventually results in neuronal injury [4]. To date, the mechanisms and treatment strategies for ischemia/hypoxia-induced neuronal damage remain elusive. Recently, numerous studies have shown that long non-coding RNAs (lncRNAs) and miRNAs are involved in mechanisms underlying ischemia/hypoxia-induced neuronal damage.

lncRNAs are approximately 200 nucleotides in length. Among lncRNAs, genomic DNA can be transcribed into RNA, but it cannot be translated into proteins [5]. lncRNAs play crucial roles in several biological processes, including the regulation of the cell cycle and differentiation and epigenetic regulation [6,7]. The roles of various lncRNAs in different diseases have been extensively reported. Several studies have shown that lncRNA expression is upregulated or downregulated in several diseases, including cancer, neurological diseases, and diabetes [8,9]. WT1 antisense RNA (WT1-AS) encodes a zinc finger transcription domain [10]. Emerging evidence has shown that WT1-AS is involved in the onset of numerous types of cancers, including breast cancer, non-small-cell lung cancer, cervical cancer, and glioma [11,12,13,14]. This study aimed to investigate the role of WT1-AS in cerebral ischemic stroke.

miRNA is a general term for a class of small non-coding RNAs, ∼20–22 nucleotides in length, that are not translated into proteins and inhibit the expression of their target genes [15]. microRNA-186-5p (miR-186-5p), a cancer-related miRNA, is involved in the occurrence and development of several types of cancers [16,17]. Zhu et al. [16] showed that miR-186-5p acts as a tumor suppressor gene that is downregulated in neuroblastoma. In addition, Jones et al. [17] demonstrated that miR-186-5p silencing inhibits the proliferation, growth, and invasion of metastatic cancer cells. Tao et al. [18] indicated that miR-186-5p may be involved in the development of atherosclerosis. However, the role of miR-186-5p in cerebral ischemic stroke has not yet been investigated. Therefore, the current study aimed to investigate whether lncRNA WT1 antisense RNA (lncRNA WT1-AS), miR-186-5p, and X-linked inhibitor of apoptosis (XIAP) could interact with each other and to uncover the underlying mechanism of action of these molecules in cerebral ischemic stroke.

## 2 Methods

### 2.1 Clinical samples

Blood samples were obtained from 30 patients with ischemic stroke within 3 h of stroke onset. In addition, 30 blood samples collected from healthy volunteers served as the control group. The characteristics of patients are shown in Table 1. The levels of lncRNA WT1-AS and miR-186-5p in the plasma of patients with ischemic stroke and healthy volunteers were determined using reverse transcription-quantitative PCR (RT-qPCR). Inclusion criteria were as follows: patients who demonstrated new-onset cerebral infarction on magnetic resonance imaging within 3 h from the time of admission were included in the study. The exclusion criteria were evidence of prior cerebral infarcts, diabetes mellitus, coronary artery disease, hypertension, kidney diseases, circulatory disorders, or autoimmune diseases. The study protocol was approved by the Ethics Committee of Taizhou People’s Hospital. All the patients in the current study approved the use of their specimens.

**Table 1:** Characteristics of patients

Characteristic	Healthy control	Stroke	*P* value
Age (year)	63–72	64–75	>0.05
Gender (male), *n* (%)	15 (50.0)	15 (50.0)	>0.05
BMI (kg/m^2^)	23.3 ± 0.34	25.1 ± 0.48	>0.05
Smoking, *n* (%)	6 (20.0)	5 (16.7)	>0.05
Drinking, *n* (%)	3 (10.0)	4 (13.3)	>0.05
Hypertension, *n* (%)	4 (13.4)	17 (56.7)	<0.05
Diabetes mellitus, *n* (%)	3 (10.0)	4 (13.3)	>0.05
Total cholesterol (mm)	4.25 ± 0.13	4.35 ± 0.26	>0.05
Triglycerides (mm)	1.53 ± 0.13	1.41 ± 0.12	>0.05
LDL (mm)	2.63 ± 0.15	2.70 ± 0.17	>0.05
HDL (mm)	1.14 ± 0.05	1.31 ± 0.07	>0.05
NIHSS score			
1–4		9 (30.0%)	
5–15		14 (46.7%)	
15–20		6 (20%)	
21–42		1 (3.3%)	

BMI, body mass index; HDL, high-density lipoprotein; LDL, low-density lipoprotein; NIHSS, National Institute of Health Stroke Scale.

### 2.2 Dual luciferase reporter assay

The wild-type (WT) or mutant (MUT) 3′-untranslated region (3′-UTR) of WT1-AS were subcloned into the pmiRGLO vector (cat. no. E1330; Promega Corporation) to assess the association between WT1-AS and miR-186-5p. Subsequently, 293T cells were co-transfected with miR-186-5p, control mimics, or the above plasmids. Following transfection for 48 h, luciferase activity was measured using a dual-luciferase assay kit (cat. no. E1910; Promega Corporation). To evaluate the association between miR-186-5p and XIAP, XIAP-WT and XIAP-MUT 3′-UTR luciferase reporter plasmids were constructed. Then, 293T cells were co-transfected with *Renilla* luciferase, luciferase reporter plasmids, and miR-186-5p or control mimics for 48 h. Luciferase activity was determined using a dual luciferase assay kit (Promega Corporation), according to the manufacturer’s instructions.

### 2.3 Cell culture and transfection

The neuroblastoma cell line, SH-SY5Y, was obtained from the American Tissue Culture Collection (cat. no. CRL-2266). Cells were cultured in Dulbecco’s modified Eagle’s medium (DMEM) supplemented with 10% fetal bovine serum (both from Gibco; Thermo Fisher Scientific, Inc.) in a cell culture incubator with 5% CO_2_ at 37°C. SH-SY5Y cells were then transfected with WT1-AS plasmid, control plasmid, mimic control (5′-UUCUCCGAACGUGUCACGUTT-3′; Shanghai GenePharma Co., Ltd., China), miR-186-5p mimics (5′-CAAAGAAUUCUCCUUUUGGGCU-3′; Shanghai GenePharma Co., Ltd., China), WT1-AS plasmid + mimic control, WT1-AS plasmid + miR-186-5p mimics, inhibitor control (5′-GCCUCCGGCUUCGCACCUCU-3′; Shanghai GenePharma Co., Ltd., China), miR-186-5p inhibitor (5′-AGCCCAAAAGGAGAAUUCUUUG-3′; Shanghai GenePharma Co., Ltd., China), control small interfering (si)-RNA, XIAP small-interfering RNA (siRNA), miR-186-5p inhibitor + control siRNA, or miR-186-5p inhibitor + XIAP siRNA for 48 h using Lipofectamine™ 2000 transfection reagent (cat. no. 11668019; Invitrogen; Thermo Fisher Scientific, Inc.), according to the manufacturer’s instructions. The cells were incubated for 48 h prior to subsequent experiments.

### 2.4 RT-qPCR assay

Total RNA was extracted from SH-SY5Y cells using TRIzol reagent (cat. no. 9108; Takara Bio, Inc.), following standard operating procedures. Total RNA was reverse transcribed into cDNA using a reverse transcriptase kit (cat no. R211-01; Vazyme Biotech Co., Ltd.). Subsequently, qPCR was performed using the SYBR Green PCR kit (cat. no. Q311-02; Vazyme Biotech Co., Ltd.), according to the manufacturer’s instructions. Glyceraldehyde-3-phosphate dehydrogenase (for mRNA) and uracil 6 (for miRNA) were used as endogenous controls. The relative gene expression was quantified using the 2^−ΔΔCq^ method. Primer sequences are listed in Table 2.

**Table 2:** Primer sequences for PCR

Gene name	Sequences: 5′–3′
lncRNA WT1-AS	Forward, 5′-GCCTCTCTGTCCTCTTCTTTG-3′
	Reverse, 5′-GCTGTGAGTCCTGGTGCTTA-3′
miR-186-5p	Forward, 5′-TCAAAGAATTCTCCTTTTGGGCT-3′
	Reverse, 5′-CGCTTCACGAATTTGCGTGTCAT-3′
GAPDH	Forward, 5′-ATCACTGCCACCCAGAAGAC-3′
	Reverse, 5′-TTTCTAGACGGCAGGTCAGG-3′
U6	Forward, 5′-CTCGCTTCGGCAGCACA-3′
	Reverse, 5′-AACGCTTCACGAATTTGCGT-3′
XIAP	Forward, 5′-ACCGTGCGGTGCTTTAGTT-3′
	Reverse, 5′-TGCGTGGCACTATTTTCAAGATA-3′

### 2.5 Establishment of the oxygen glucose deprivation/reoxygenation (OGD/R) cell model

To establish an *in vitro* OGD/R model, the cells were cultured in glucose-free DMEM at 37°C in an atmosphere of 1% O_2_, 94% N_2_, and 5% CO_2_ for 4 h. Subsequently, the cells were cultured under normoxic conditions of 95% air and 5% CO_2_ for an additional 24 h.

### 2.6 Western blotting analysis

The cells were lysed with the radioimmunoprecipitation assay buffer (cat. no. R0010; Beijing Solarbio Science & Technology Co., Ltd.), and the protein concentration was measured using a bicinchoninic acid kit (cat no. 23225; Pierce; Thermo Fisher Scientific, Inc.). Equal amounts of proteins were separated via 12% sodium dodecyl sulfate-polyacrylamide gel electrophoresis and transferred onto polyvinylidene fluoride membranes. Following blocking with 5% nonfat milk to prevent nonspecific binding, the membranes were incubated with primary antibodies against XIAP (cat. no. ab21278; dilution, 1:1,000) and GAPDH (cat. no. ab9485; dilution, 1:1,000; both from Abcam) at 4°C overnight. The next day, membranes were incubated with the corresponding secondary antibodies for 2 h. Protein bands were visualized using the ECL method (cat. no. 34579; Applygen Technologies, Inc.).

### 2.7 Flow cytometric assay

Cell apoptosis was evaluated using the Annexin-V-fluorescein isothiocyanate (FITC)/propidium iodide (PI) Apoptosis Detection Kit (cat. no. 556570; BD Bioscience). Briefly, after treatment, the cells were collected and centrifuged at a high speed and low temperature. Following centrifugation, the supernatant was discarded and the cells were resuspended in 100 μL FITC-binding buffer. Subsequently, the cell suspension was supplemented with 5 μL ready-to-use annexin V-FITC and 5 μL PI. The cells were incubated for 30 min at room temperature in the dark. Cell apoptosis rate was assessed using a BD FACSCalibur flow cytometer (BD Technologies).

### 2.8 3-(4,5-Dimethylthiazol-2-yl)-2,5-diphenyl tetrazolium bromide (MTT) assay

Following treatment, cells were seeded into a 96-well plate and incubated for 24 h. Subsequently, each well was supplemented with 20 μL MTT reagent (5 mg/mL; cat. no. CT02; Sigma-Aldrich), and the cells were cultured for an additional 4 h. The supernatant was discarded and each well was supplemented with 200 µL dimethyl sulfoxide. The absorbance of each well was measured at 570 nm wavelength.

### 2.9 Caspase 3 activity detection

Caspase 3 activity was assessed using the corresponding detection kit (cat. no. C1116; Beyotime Institute of Biotechnology). Briefly, transfected cells were collected into tubes and centrifuged at 600×*g* for 5 min at 4°C. The cells were resuspended in an appropriate volume of lysis buffer and lysed for 15 min in an ice bath. Following centrifugation for 10 min, the supernatant was transferred to a pre-cooled centrifuge tube and incubated on ice. The enzymatic activity of caspase 3 was measured immediately at a wavelength of 405 nm.

### 2.10 Statistical analysis

All experiments were repeated at least three times. All data were analyzed using GraphPad Prism software (version 6.0; GraphPad Software, Inc.). Statistically significant differences between two groups were evaluated using unpaired Student’s *t*-test, while those among multiple groups were evaluated using one-way analysis of variance followed by Tukey’s *post hoc* test. Data are expressed as the mean ± standard deviation from three independent experiments. *P* < 0.05 was considered to be statistically significant.

## 3 Results

### 3.1 lncRNA WT1-AS directly interacts with miR-186-5p

**Figure 1:**
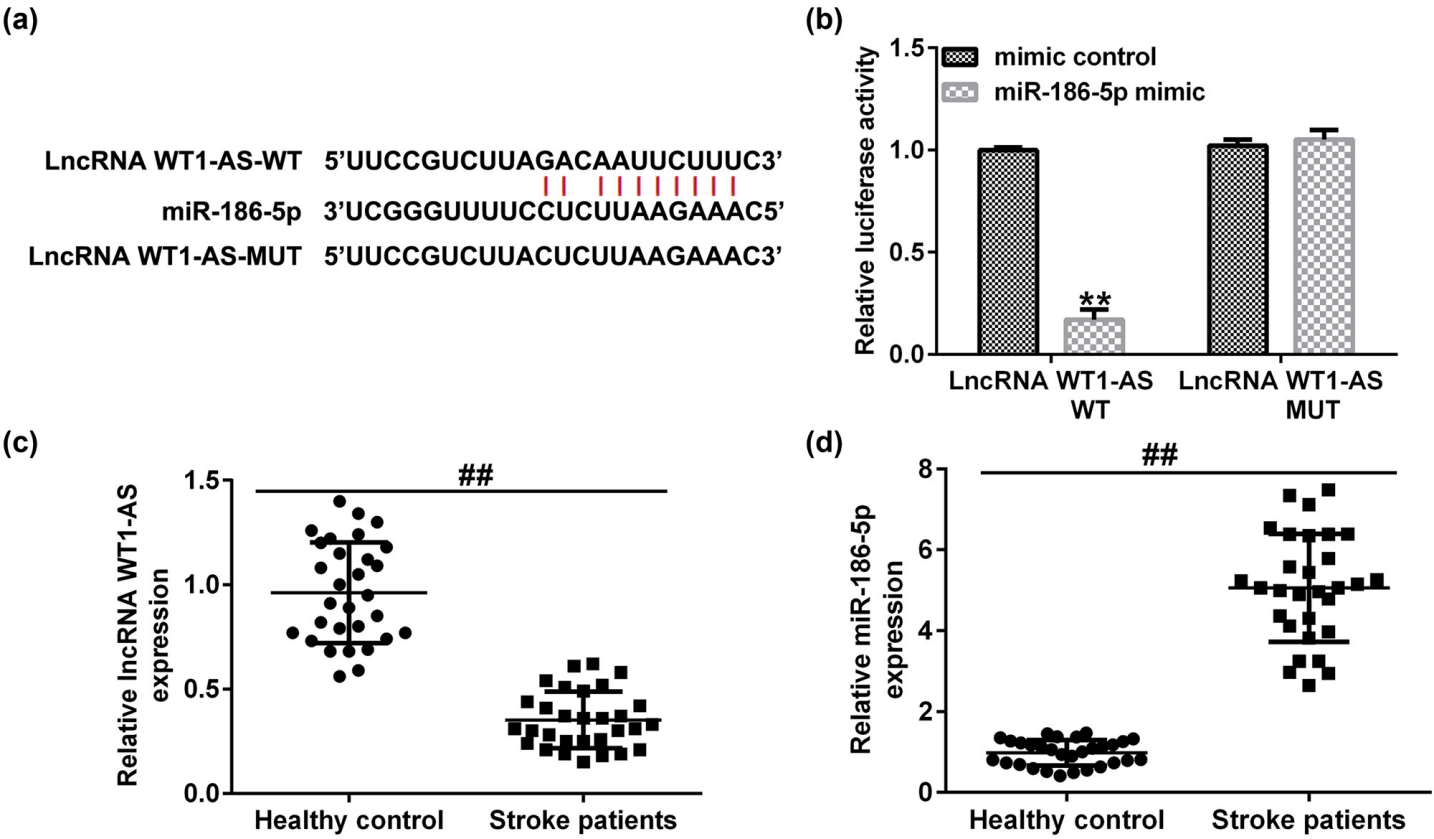
Expression levels of lncRNA WT1-AS and miR-186-5p in patients with ischemic stroke. (a) Interaction between miR-186-5p and WT1-AS 3′-UTR was predicted using the Starbase prediction software. (b) Dual luciferase reporter gene assay was used to verify the interaction between WT1-AS and miR-186-5p in 293T cells co-transfected with miR-186-5p mimics and WT or MUT WT1-AS 3′-UTR reporter plasmids. (c and d) Expression levels of WT1-AS and miR-186-5p in blood samples of patients with ischemic stroke and healthy individuals. ^**^ *P* < 0.01 vs control mimics group; ^##^ *P* < 0.01 vs healthy control group. GraphPad Prism 6.0 software (GraphPad Software, Inc.) was used for creation of the figure.

First, the association between WT1-AS and miR-186-5p was predicted using the StarBase bioinformatics tool. Bioinformatics analysis revealed that miR-186-5p and lncRNA WT1-AS shared mutual binding sites (Figure 1a), suggesting that miR-186-5p could bind to the WT1-AS 3′-UTR. Subsequently, 293T cells were cotransfected with WT1-AS-WT, WT1-AS-MUT, miR-186-5p, or mimic control for 48 h. A dual-luciferase reporter assay was then performed to evaluate the luciferase activity. The results revealed that miR-186-5p mimics inhibited the activity of WT1-AS-WT but not that of WT1-AS-MUT (Figure 1b). These findings indicate that miR-186-5p is directly targeted by the lncRNA WT1-AS. Subsequently, blood samples from 30 patients with ischemic stroke were collected within 3 h of stroke onset. RT-qPCR assay results demonstrated that, compared with the healthy control group, lncRNA WT1-AS was substantially downregulated (Figure 1c) and miR-186-5p was upregulated (Figure 1d) in the blood samples of patients with ischemic stroke.

### 3.2 lncRNA WT1-AS levels are downregulated and miR-186-5p levels are upregulated in OGD-induced SH-SY5Y cells

**Figure 2:**
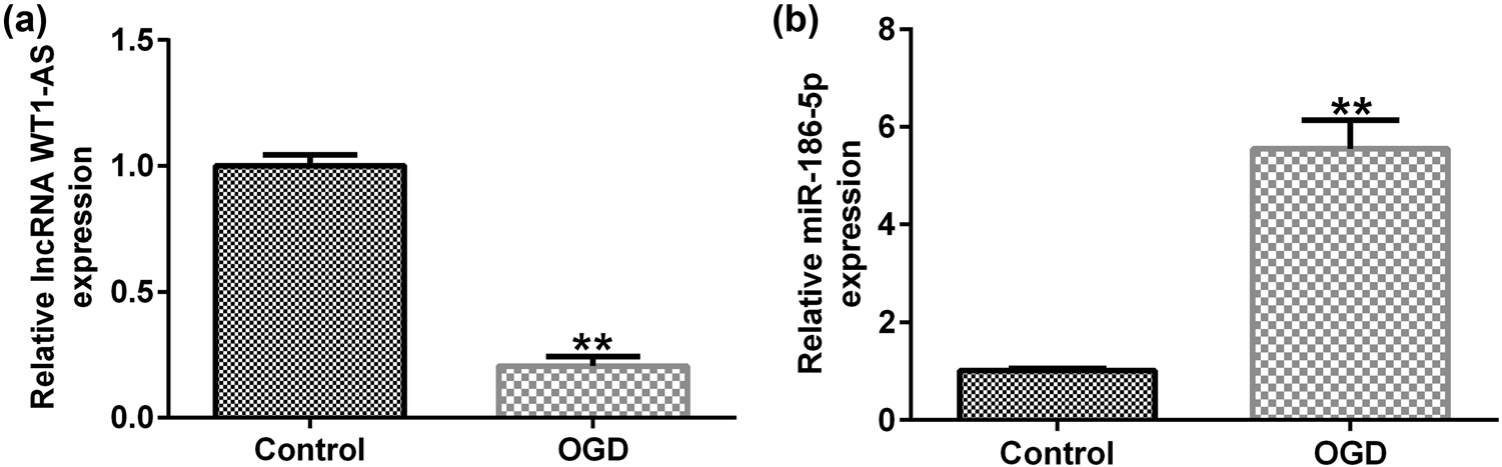
Expression levels of lncRNA WT1-AS/miR-186-5p in OGD-induced SH-SY5Y cells. (a) RT-qPCR analysis results showing lncRNA WT1-AS expression levels. (b) RT-qPCR analysis results showing miR-186-5p expression levels. ^**^ *P* < 0.01 vs control group. GraphPad Prism 6.0 software (GraphPad Software, Inc.) was used for creation of the figure.

The results showed that, compared with the control group, the expression of lncRNA WT1-AS was significantly reduced in OGD-induced SH-SY5Y cells (Figure 2a), while miR-186-5p expression was significantly upregulated (Figure 2b).

### 3.3 WT1-AS negatively regulates miR-186-5p levels in SH-SY5Y cells

**Figure 3:**
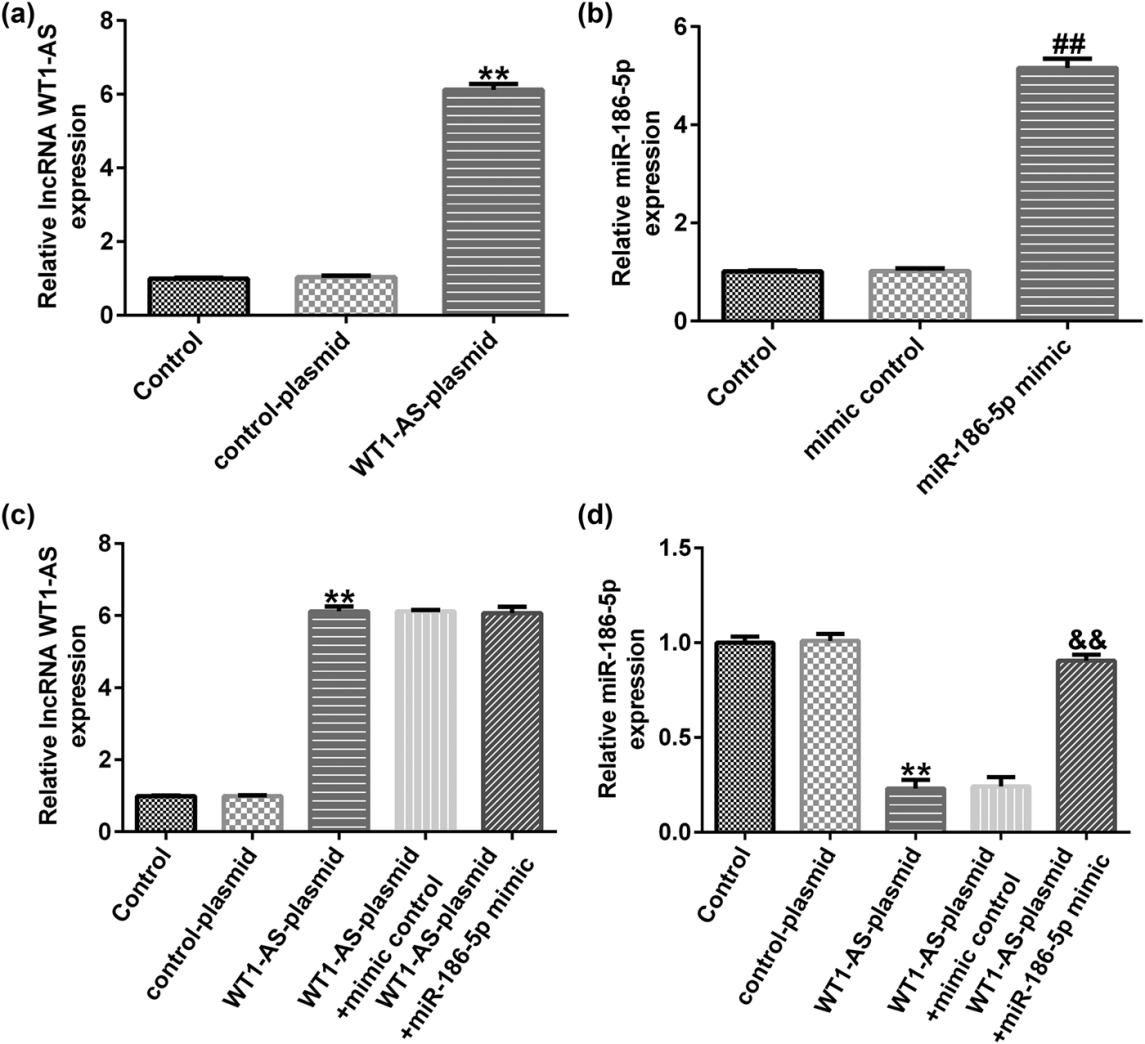
lncRNA WT1-AS negatively regulates miR-186-5p expression levels in SH-SY5Y cells. (a) RT-qPCR analysis results showing WT1-AS expression levels in SH-SY5Y cells transfected with the control or WT1-AS-plasmids. (b) RT-qPCR analysis results showing miR-186-5p expression levels in SH-SY5Y cells transfected with the control or miR-186-5p mimics. (c) RT-qPCR analysis showing the expression levels of WT1-AS in SH-SY5Y cells transfected with the control plasmid, miR-186-5p mimics, WT1-AS plasmid + control mimics, or WT1-AS plasmid + miR-186-5p mimics. (d) RT-qPCR analysis showing the expression levels of miR-186-5p in SH-SY5Y cells transfected with the control plasmid, miR-186-5p mimics, WT1-AS plasmid + control mimics, or WT1-AS plasmid + miR-186-5p mimics. ^**^ *P* < 0.01 vs control plasmid group; ^##^ *P* < 0.01 vs control mimics group; ^&&^ *P* < 0.01 vs WT1-AS plasmid + control mimics group. GraphPad Prism 6.0 software (GraphPad Software, Inc.) was used for creation of the figure.

Compared with the control plasmid group, cell transfection with the WT1-AS plasmid successfully overexpressed WT1-AS in SH-SY5Y cells (Figure 3a). In addition, compared to the mimic control group, transfection with miR-186-5p mimics notably increased miR-186-5p expression in SH-SY5Y cells (Figure 3b). There were no significant changes in the expression of WT1-AS in WT1-AS plasmid + mimic control and WT1-AS plasmid + miR-186-5p mimics groups (Figure 3c). Finally, WT1-AS overexpression inhibited miR-186-5p expression compared to the control plasmid group, while this effect was notably reversed by miR-186-5p overexpression (Figure 3d). The data indicated that WT1-AS negatively regulates miR-186-5p levels in SH-SY5Y cells.

### 3.4 lncRNA WT1-AS attenuates OGD-induced neuronal injury by downregulating miR-186-5p levels

**Figure 4:**
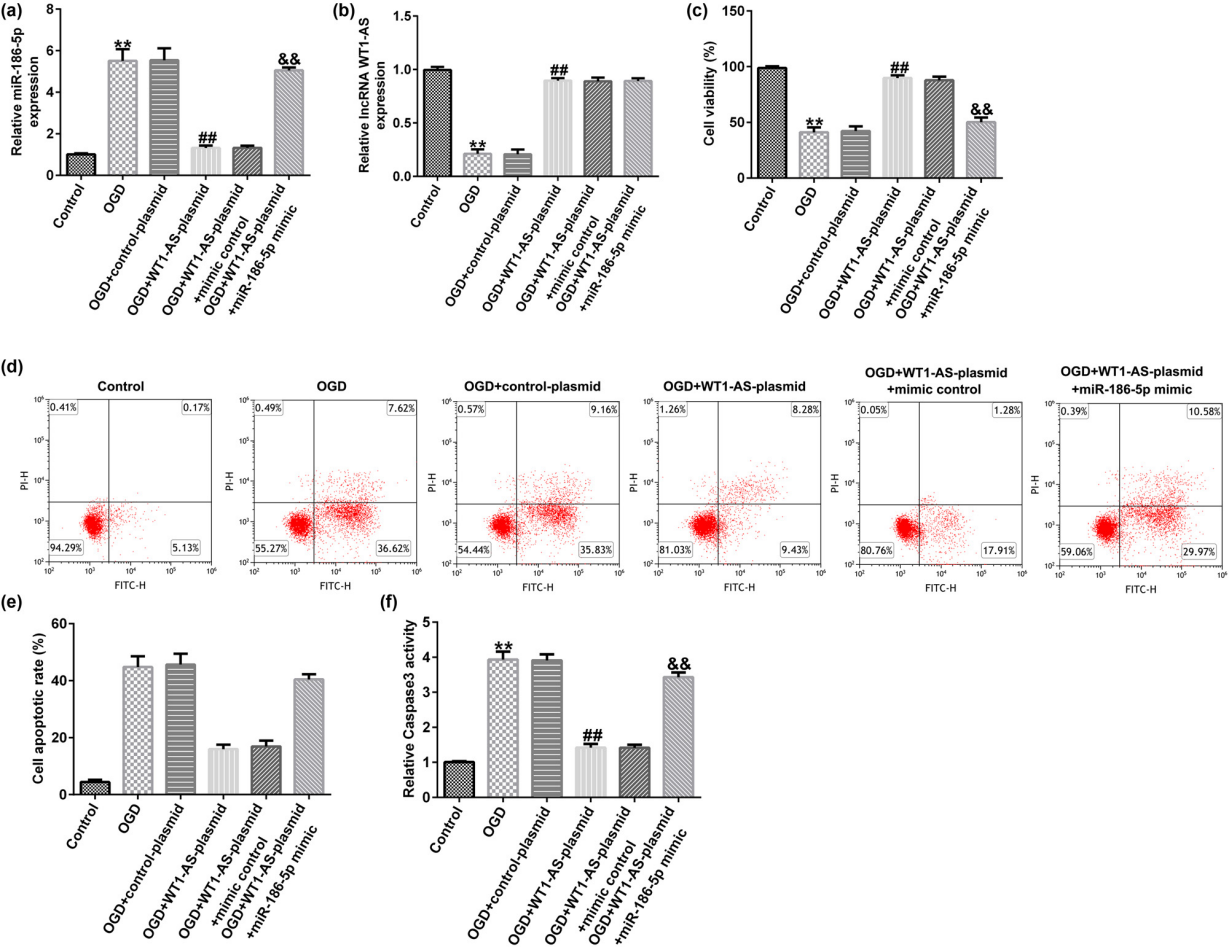
Effect of lncRNA WT1-AS on OGD-induced nerve cell injury. (a) RT-qPCR analysis results showing microRNA-186-5p expression levels. (b) RT-qPCR analysis results showing lncRNA WT1-AS expression levels. (c) A MTT assay was used to assess the cell viability. (d) A flow cytometric assay was used to evaluate cell apoptosis. (e) The apoptosis ratio is presented. (f) Caspase 3 activity was detected using the corresponding kit. ^**^ *P* < 0.01 vs control group; ^##^ *P* < 0.01 vs OGD + control mimics group; ^&&^ *P* < 0.01 vs OGD + WT1-AS plasmid + control mimics group. GraphPad Prism 6.0 software (GraphPad Software, Inc.) was used for creation of the figure.

Compared to the control group, miR-186-5p expression was significantly enhanced in the OGD group. In addition, the expression of miR-186-5p was significantly reduced in the OGD + WT1-AS plasmid group compared to that in the OGD + control plasmid group. However, this effect was reversed by miR-186-5p overexpression (Figure 4a). Furthermore, RT-qPCR analysis showed that compared to the control group, the expression of lncRNA WT1-AS decreased in the OGD group. In addition, compared to the OGD + control plasmid group, lncRNA WT1-AS was upregulated in the OGD + WT1-AS plasmid group (Figure 4b). MTT assay and flow cytometric analysis revealed that compared to the control group, cell viability decreased (Figure 4c) and cell apoptosis increased (Figure 4d and e), respectively, in the OGD group. Compared to the control group, the activity of caspase 3 was enhanced in the OGD group (Figure 4f). Compared to the OGD + control plasmid group, cell viability was significantly increased (Figure 4c), apoptosis (Figure 4d and e) was reduced, and caspase 3 activity was attenuated (Figure 4f) in the OGD + WT1-AS plasmid group. Furthermore, all the aforementioned effects caused by WT1-AS plasmid were reversed following miR-186-5p overexpression. These findings suggested that lncRNA WT1-AS attenuates OGD-induced neuronal injury by downregulating miR-186-5p levels.

### 3.5 miR-186-5p directly interacts with XIAP

**Figure 5:**
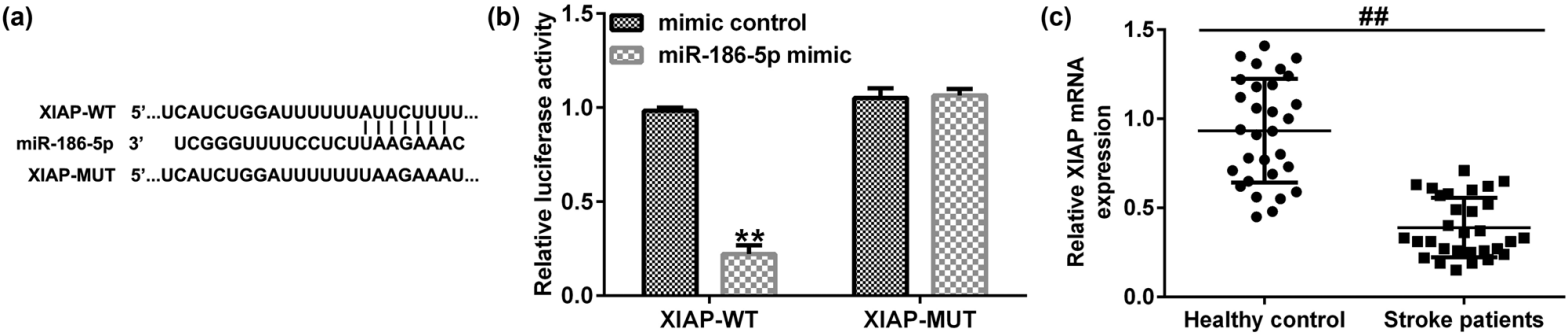
XIAP is directly targeted by miR-186-5p. (a) The interaction between miR-186-5p and XIAP 3′-UTR was predicted by TargetScan. (b) A dual luciferase reporter gene assay was performed to verify the interaction between miR-186-5p and XIAP in 293T cells co-transfected with miR-186-5p mimics and WT or MUT XIAP 3′-UTR reporter plasmids. (c) Expression levels of XIAP in blood samples of patients with ischemic stroke and healthy individuals.^**^ *P* < 0.01 vs control mimics group; ^##^ *P* < 0.01 vs Healthy control group. GraphPad Prism 6.0 software (GraphPad Software, Inc.) was used for creation of the figure.

A literature review [19] and bioinformatic analysis using the TargetScan database identified a mutual binding site between miR-186-5p and XIAP (Figure 5a). Furthermore, a dual-luciferase reporter assay verified the association between miR-186-5p and XIAP. As shown in Figure 5b, miR-186-5p mimics inhibited the activity of XIAP-WT but not that of XIAP-MUT. Then, we determined the level of XIAP in the blood samples of patients with ischemic stroke. The results indicated that compared with the healthy control group, XIAP was significantly downregulated (Figure 5c) in the blood samples of patients with ischemic stroke. Taken together, these findings suggest that XIAP is directly targeted by miR-186-5p, and it is downregulated in the blood samples of patients with ischemic stroke.

### 3.6 miR-186-5p negatively regulated XIAP expression in SH-SY5Y cells

**Figure 6:**
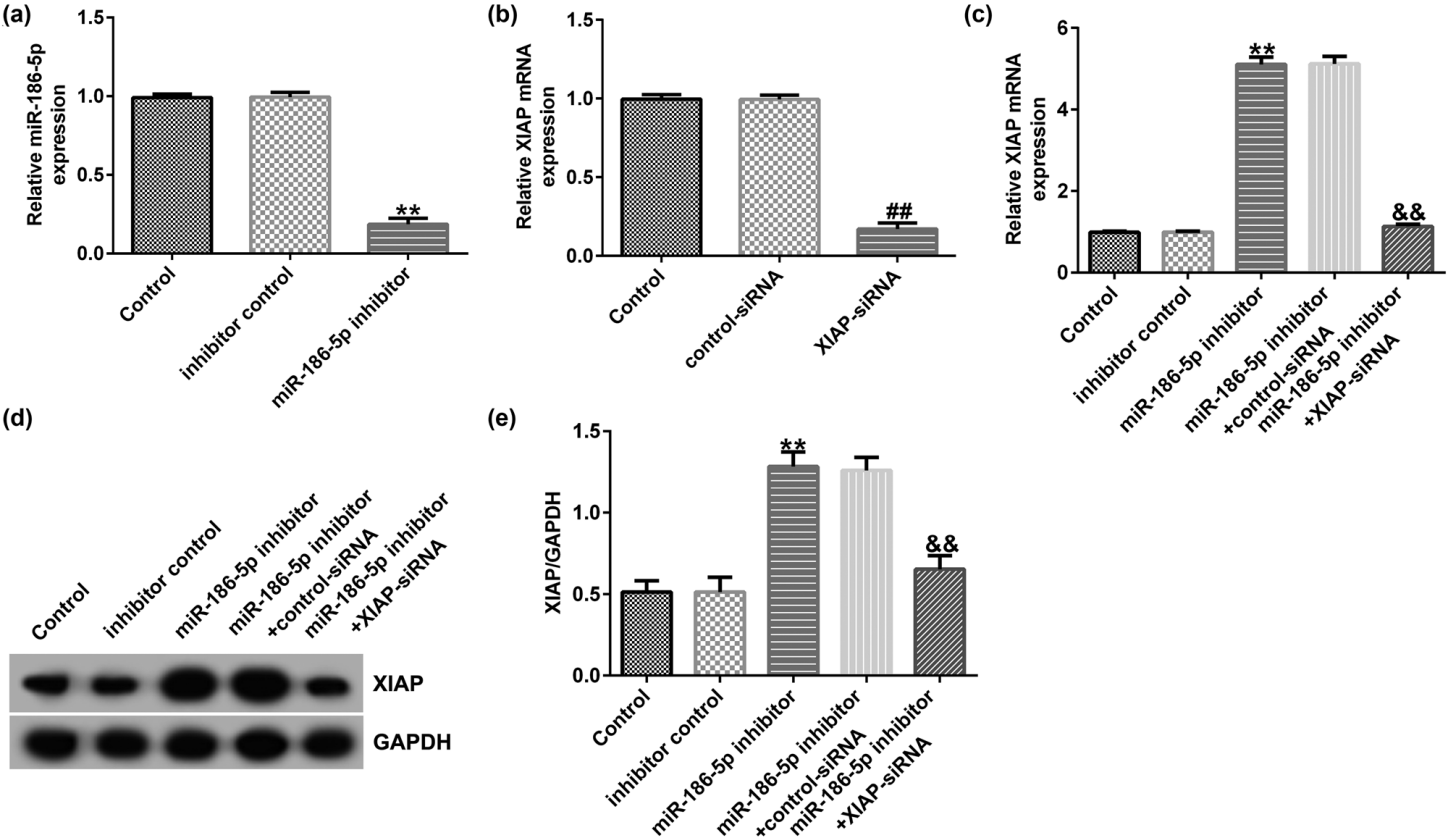
miR-186-5p negatively regulates XIAP expression levels in SH-SY5Y cells. (a) RT-qPCR assay results showing miR-186-5p expression levels in SH-SY5Y cells transfected with the inhibitor control or miR-186-5p inhibitors. (b) RT-qPCR assay results showing XIAP expression levels in SH-SY5Y cells transfected with the control or XIAP siRNAs. (c) RT-qPCR assay results showing XIAP expression levels in SH-SY5Y cells transfected with the miR-186-5p inhibitor + control siRNA or miR-186-5p inhibitor + XIAP siRNA. (d) Western blotting analysis results showing the protein expression levels of XIAP. (e) XIAP/GAPDH. ^**^ *P* < 0.01 vs control inhibitor group; ^##^ *P* < 0.01 vs control siRNA group; ^&&^ *P* < 0.01 vs miR-186-5p inhibitor + control siRNA group. GraphPad Prism 6.0 software (GraphPad Software, Inc.) was used for creation of the figure.

SH-SY5Y cells were transfected with inhibitor control, miR-186-5p inhibitor, control siRNA, XIAP siRNA, miR-186-5p inhibitor + control siRNA, or miR-186-5p inhibitor + XIAP siRNA for 24 h. RT-qPCR was performed to assess the transfection efficiency. Compared to the inhibitor control group, transfection with the miR-186-5p inhibitor significantly reduced miR-186-5p expression in SH-SY5Y cells (Figure 6a). Consistently, compared to the control siRNA group, transfection with XIAP siRNA notably attenuated XIAP expression (Figure 6b). However, miR-186-5p silencing significantly increased the mRNA and protein expression levels of XIAP compared to those in the control inhibitor group, and this effect was reversed by XIAP knockdown (Figure 6c–e). These findings suggest that XIAP is negatively regulated by miR-186-5p in SH-SY5Y cells.

### 3.7 Effect of miR-186-5p silencing on OGD-induced nerve cell injury

**Figure 7:**
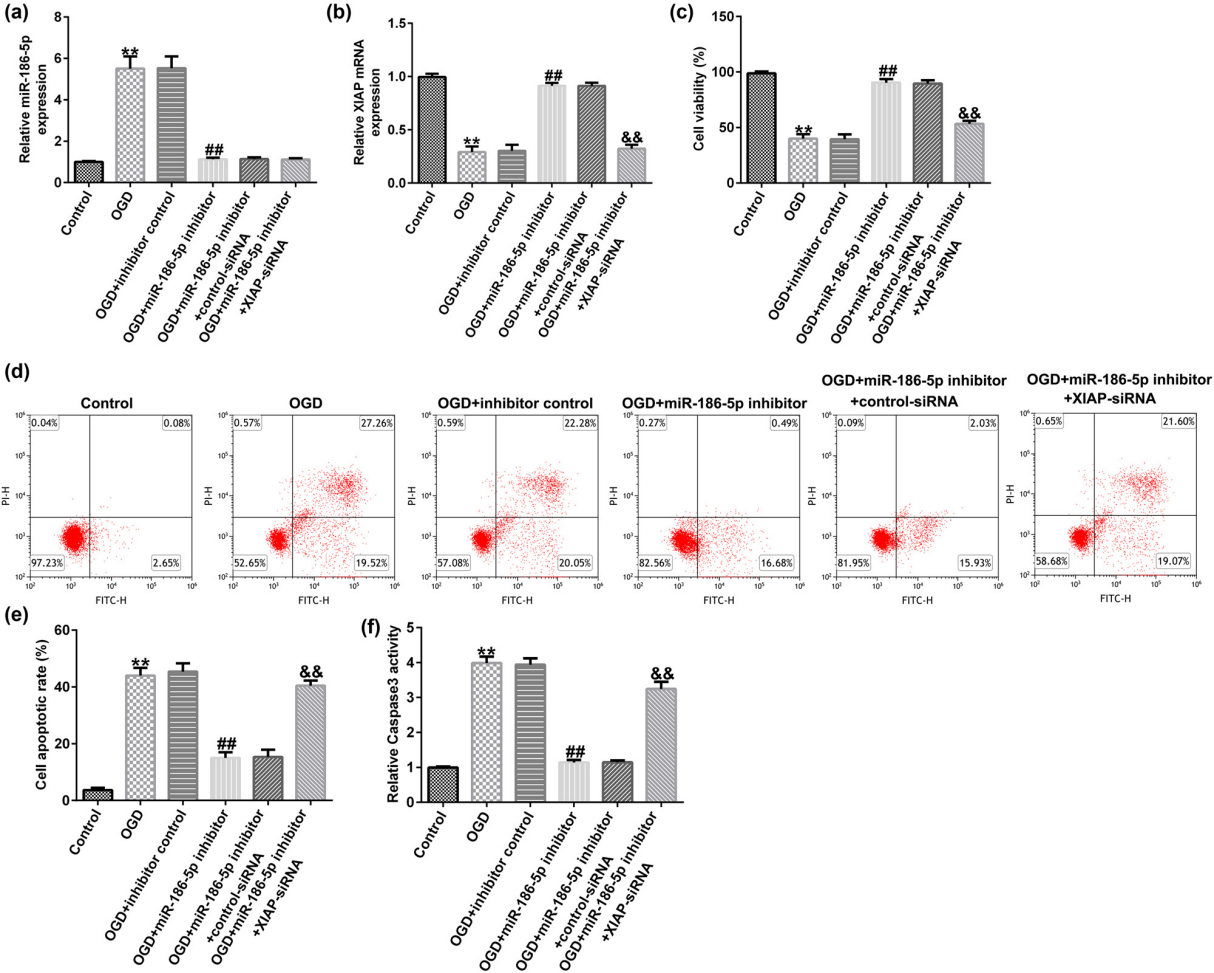
miR-186-5p inhibitor attenuates OGD-induced neuronal injury by upregulating XIAP expression. (a) RT-qPCR analysis results showing miR-186-5p expression levels. (b) RT-qPCR analysis results showing XIAP expression levels. (c) MTT assay was used to assess the cell viability. (d) Flow cytometric assay was carried out to evaluate cell apoptosis. (e) The cell apoptosis ratio is presented. (f) Caspase 3 activity was detected using the corresponding kit. ^**^ *P* < 0.01 vs control group; ^##^ *P* < 0.01 vs OGD + inhibitor control group; ^&&^ *P* < 0.01 vs OGD + miR-186-5p inhibitor + control siRNA group. GraphPad Prism 6.0 software (GraphPad Software, Inc.) was used for creation of the figure.

Following cell transfection with inhibitor control, miR-186-5p inhibitor, miR-186-5p inhibitor + control-siRNA, or miR-186-5p inhibitor + XIAP-siRNA for 24 h, an OGD-induced cell injury model was established in SH-SY5Y cells. The results demonstrated that miR-186-5p levels were upregulated (Figure 7a) and XIAP levels were downregulated (Figure 7b) in the OGD group compared to those in the control group. In addition, compared with the OGD + inhibitor control group, the expression of miR-186-5p and XIAP decreased and increased, respectively, in the OGD + miR-186-5p inhibitor group. Finally, compared with the OGD + inhibitor control group, cell viability was improved (Figure 7c) and cell apoptosis (Figure 7d and e) and caspase 3 activity (Figure 7f) were reduced in the OGD + miR-186-5p inhibitor group, and all these effects were reversed by the knockdown of XIAP. In general, miR-186-5p silencing relieves OGD-induced nerve cell injury via up-regulating XIAP expression.

## 4 Discussion

Acute cerebral ischemic stroke is characterized by an increased mortality rate in humans and is commonly accompanied by several complications, such as behavioral, social, attention, cognitive, and functional motor dysfunctions [20,21]. The pathological process of ischemic stroke is complicated and the factors contributing to the disease are diverse. Neuroinflammation and oxidative stress have been reported to play significant roles in ischemic brain injury [22,23]. Although several treatment strategies have been previously applied to treat ischemic stroke, the therapeutic potential of currently available treatment approaches remains unsatisfactory [24,25,26]. Therefore, novel treatment methods for ischemic stroke are urgently required.

The current study aimed to investigate the role of the lncRNA WT1-AS in cerebral ischemic stroke. The roles of lncRNAs in several diseases, including cerebral ischemic stroke, have attracted increasing attention. Shan et al. [27] showed that the lncRNA taurine-upregulated gene 1 (TUG1) is involved in the development of cerebral ischemic stroke, and TUG1 silencing can diminish OGD/R-induced injury. Another study by Wen et al. [28] indicated that lncRNA Gm4419 overexpression upregulates TNF-α, IL-1β, and IL-6 levels, thereby enhancing OGD/R damage. Hu et al. [29] showed that knockdown of lncRNA small nucleolar RNA host gene 15 could improve ischemia/hypoxia-induced neuronal injury and microglial inflammation by targeting the miR-302a-3p/signal transducer and activator of transcription 1 axis. Furthermore, Xiao et al. [30] demonstrated that lncRNA H19 was significantly upregulated in patients with ischemic stroke, whereas its silencing could attenuate neuronal apoptosis in OGD-induced neuronal cells by targeting miR-19a. In this study, bioinformatics analysis and dual-luciferase reporter assays were performed to verify the interaction between lncRNA WT1-AS and miR-186-5p.

lncRNA WT1-AS is involved in cancer development [31,32,33]. To the best of our knowledge, the effects of lncRNA WT1-AS on ischemic stroke have not yet been reported. Consistent with previous studies, the results of the present study demonstrated that WT1-AS levels were downregulated and miR-186-5p levels were upregulated in the blood samples of patients with ischemic stroke. Similarly, the expression levels of WT1-AS and miR-186-5p were enhanced and reduced, respectively, in OGD-treated SH-SY5Y cells *in vitro*.

Subsequently, the mechanisms underlying the effects of WT1-AS and miR-186-5p on OGD-induced SH-SY5Y cell injury were investigated. Du et al. [34] showed that LINC01705 was directly targeted by miR-186-5p and was involved in breast cancer development. In addition, Zhu et al. [35] demonstrated that HLA complex P5 could promote neuroblastoma cell proliferation by downregulating miR-186-5p expression. In osteosarcoma, miR-186-5p attenuates cell proliferation, invasion, and metastasis by targeting Forkhead Box K1 [36]. In this study, WT1-AS overexpression attenuated OGD-induced neuronal injury by downregulating the expression of miR-186-5p. Furthermore, the results of the current study revealed that XIAP was a direct target of miR-186-5p.

XIAP, a member of the inhibitor of apoptosis protein family, is a cytoplasmic inhibitor of caspases 3, 7, and 9 [37]. As a target for cancer treatment, XIAP is abnormally expressed in cancer, thus playing a significant role in regulating patient mortality [38]. Huang et al. [39] revealed that miR-377-3p can suppress colorectal cancer by regulating XIAP expression. Deng et al. [40] demonstrated that XIAP is involved in the development of ischemic stroke, and its expression is reduced in middle cerebral artery occlusion (MCAO) model rats. Therefore, XIAP silencing can reverse the effects of miR-130a downregulation on neurological function and angiogenesis in MCAO model rats. In this study, miR-186-5p silencing alleviated the OGD-induced SH-SY5Y cell injury. Besides, activation of caspase3 was found to be mechanism of XIAP silencing-induced apoptosis. Since caspase3 is activated by proteolytic cleavage, total caspase3 and cleaved caspase3 levels could be determined. This study did not analyze the total caspase3 and cleaved caspase3 level, which was a limitation of this study. Overall, the results of the present study indicate that WT1-AS plays a crucial role in cerebral ischemic stroke by regulating the miR-186-5p/XIAP axis. lncRNA WT1-AS attenuates hypoxia/ischemia-induced neuronal injury in cerebral ischemic stroke via the miR-186-5p/XIAP axis.

However, to further elucidate the role of lncRNA WT1-AS in ischemic stroke, future in-depth studies are needed. For example, more cell lines may be required to confirm that the effect of IncRNA WT1-AS on neuronal damage is general activity and not SH-SY5Y specific. The effects of XIAP over-expression on OGD induced neuronal injury should be further clarified. Besides, the role of lncRNA WT1-AS in ischemic stroke should be explored using animal models. In addition, the correlation between lncRNA WT1-AS expression and clinicopathological parameters of ischemic stroke patients should be investigated. These issues will be addressed in the future.

## 5 Conclusion

In this study, we found that lncRNA WT1-AS reduced OGD-induced SH-SY5Y cell injury via the miR-186-5p/XIAP axis, indicating the protective role of lncRNA WT1-AS in hypoxia/ischemia-induced neuronal injury in cerebral ischemic stroke.

## Abbreviations

FITC: fluorescein isothiocyanate
lncRNA: long non-coding RNA
MTT: 3-(4,5-dimethylthiazol-2-yl)-2,5-diphenyl tetrazolium bromide
OGD: oxygen glucose deprivation
PI: propidium iodide
RT-qPCR: reverse transcription-quantitative PCR
WT1-AS: WT1 antisense RNA
XIAP: X-linked inhibitor of apoptosis
